# Duodenal perforation in a 12-month old child with severe malaria

**Published:** 2012-05-03

**Authors:** Nina Goldman, Damien Punguyire, Kingsley Osei-Kwakye, Frank Baiden

**Affiliations:** 1 University of Cambridge, School of Clinical Medicine, Addenbrooke's Hospital, Hills Road, Cambridge, CB2 0SP, UK; 2 Kintampo Municipal Hospital, Po Box 192, Kintampo, BA Ghana; 3 Kintampo Health Research Centre, PO Box 200, Kintampo, BA Ghana

**Keywords:** Peptic ulcer disease, malaria, children, intestinal perforation

## Abstract

Peptic ulcer disease (PUD) in children remains rare and difficult to diagnose before the onset of complications. We report on a case of a 12-month child with perforated duodenal ulcer, association with malaria. The severity of the febrile presentation and the positive laboratory confirmation of malaria delayed the diagnosis of PUD. Surgical intervention was successful and without significant sequelae. An awareness of the possibility, and a lower threshold for considering PUD in children may help prevent complications.

## Introduction

Peptic ulcer disease (PUD) is uncommon and difficult to diagnose in young children and clinical features are often non-specific [1]. We report a case of a 12-month old boy with severe malaria who developed duodenal perforation.

## Patient and case report

A previously healthy 12-month old boy was referred from a clinic to the Kintampo Municipal Hospital (Kintampo, Ghana). He had presented at the clinic with a 2-day history of fever and vomiting. On admission to the clinic, diagnosis of severe anaemia secondary to Malaria was made. Despite treatment with IV fluid, IM Quinine and a blood transfusion, his condition deteriorated and he began to pass bloody stools. After two days he was referred to hospital for further management.

On arrival, the child had a reduced conscious level. He was febrile (39.5°C) and pale with pulse and respiration rates 110 beat/min and 60 breaths/min respectively. Abdominal examination was normal apart from periumbilical tenderness.

Blood film was positive for malaria parasites. Haemoglobin was 4.2g/dL and white blood cell count 16.4 × 10^9^. Neither Widal test nor stool cultures were done. Supine abdominal X-ray showed air fluid levels. Erect chest X-ray showed no subphrenic gas. Treatment of severe anaemia secondary to malaria and suspected typhoid was continued. Intranasal oxygen, IV fluids and whole blood were administered along with paracetamol, IM quinine, IV gentamicin and IV metronidazole. After a further 2 days he remained semiconscious and febrile, passing blood clots in his stool. Despite transfusion, haemoglobin remained 4.3g/dL. Abdominal examination remained unremarkable.

**Figure 1:**
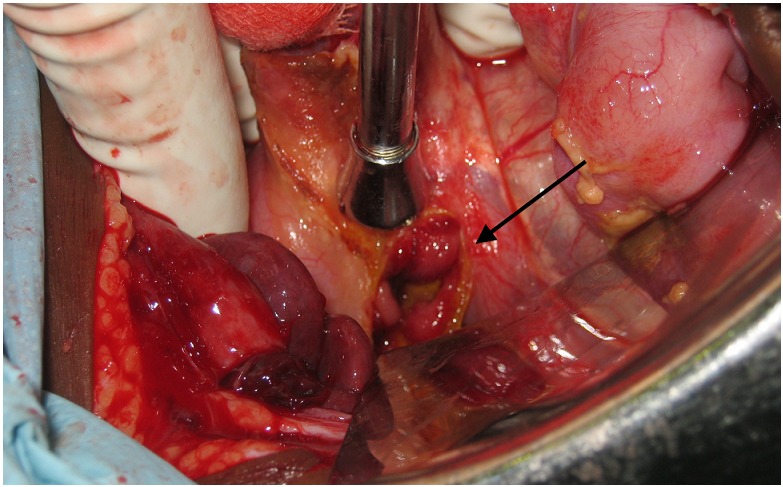
1.5 cm anterior duodenal wall perforation (arrowed) in a 12-month old child with severe malaria.

An exploratory laparotomy was performed. On entering the peritoneum a large amount of blood-stained fluid was seen. A 1.5cm anterior perforation of the duodenal wall was found, with bleeding from the posterior duodenal wall (Figure 1). The bleeding vessel was clamped and the perforation closed using an omental patch.

Postoperatively the child received a further blood transfusion, antibiotics and IV ranitidine. Ten days post-operatively he was discharged. On follow-up he had no sequelae.

Due to resource constraint no test for *Helicobacter pylori* was performed. The mother denied giving any medication to the child.

## Discussion

PUD is rare in children with an estimated frequency of 1 in 3000 hospital admissions [1]. Symptoms are often non-specific and difficult to assess. Endoscopy has improved diagnosis in suspected PUD but is often not available in resource poor settings, making diagnosis challenging [2].

PUD in children is classified as primary or secondary depending on the aetiology. Secondary PUD is associated with physiological stress, systemic illness and drugs including non-steroidal anti-inflammatories and steroids. Primary PUD is commonly associated with Helicobacter pylori infection. It can also be idiopathic or associated with conditions causing increased acid secretion [1, 3].

PUD is more commonly secondary in children under ten years of age and primary in the over tens. Secondary PUD occurs in the stomach or duodenum and is more likely to present with complications including perforation and hemorrhage. Primary PUD predominantly occurs in the duodenum and rarely presents with perforation [4].

94% of children with perforated PUD present with acute abdominal signs [5]. Free air under the diaphragm is found in 82.7% [5]. Surgery is the recommended treatment for perforation. Medical management of secondary PUD includes histamine receptor blockers and elimination of physiological stress. If treated secondary PUD rarely recurs [2].

A literature search revealed no reports of perforated duodenal ulcers associated with malaria. In our case, we postulate that physiological stress from severe malaria resulted in secondary PUD progressing to perforation due to characteristics of secondary PUD and diagnostic challenges.

## Conclusion

PUD remains difficult to diagnose in children prior to complications especially in a resource poor settings. A lower threshold for considering PUD in children may help prevent complications.
